# Gross anatomy of the longitudinal fascicle of *Sapajus* sp.

**DOI:** 10.1371/journal.pone.0252178

**Published:** 2021-06-24

**Authors:** Tales Alexandre Aversi-Ferreira, Kellen Christina Malheiros Borges, Maria Tereza Gonçalves-Mendes, Leonardo Ferreira Caixeta

**Affiliations:** 1 Department of Structural Biology, Laboratory of Biomathematics and Physical Anthropology, Institute of Biomedical Science, Federal University of Alfenas, Alfenas, Minas Gerais, Brazil; 2 Department of Biology, Academic Areas, Federal Institute of Goias, Anápolis, Goiás, Brazil; 3 Department of Behavioral Neurology, Clinical Hospital, Federal University of Goias, Goiânia, Goiás, Brazil; Federal University of Rio Grande do Norte, BRAZIL

## Abstract

Opposing genetic and cultural-social explanations for the origin of language are currently the focus of much discussion. One of the functions linked to the longitudinal fascicle is language, which links Wernicke’s area and Broca’s area in the brain, and its size should indicate the brain increase in the evolution. *Sapajus* is a New World primate genus with high cognition and advanced tool use similar to that of chimpanzees. A study of the gross anatomy of the longitudinal fascicle of *Sapajus* using Kingler’s method found it to differ from other studied primates, such as macaques and chimpanzees, mainly because its fibers join the cingulate fascicle. As in other non-human primates, the longitudinal fascicle of *Sapajus* does not reach the temporal lobe, which could indicate a way of separating these fascicles to increase white matter in relation to individual function. The study of anatomical structures seems very promising for understanding the basis of the origin of language. Indeed, socio-historical-cultural philosophy affirms the socio-cultural origin of speech, although considering the anatomical structures behind it working as a functional system.

## Introduction

A new hypothesis about the origin of language has recently been proposed [1], in opposition, in many respects, to current genetic theory [2–4]. The origin of language from a natural human capacity has allowed the construction of an infinity of phrases from a finite number of words and limited grammatical rules [2]; therefore, this natural human capacity has a genetic basis. On the other hand, the development of human social and cultural structure seems to have had an important role in the development of language [1], considering study of the Pirahã language, inter alia [5].

It must be assumed that language, like other higher cortical functions, did not appear suddenly in humans, but is based on the convergence of human invention, cognition and psychological evolution [1], in addition to environmental pressure.

The cultural importance of the evolution of speech is not a new approach. In fact, the socio-historic-cultural philosophy developed by Russian psychologists in the first half of the 20^th^ Century considered human cognition to be strongly dependent on language and its neural physiological substrate [6].

However, phylogenetic considerations about the basis of language were not completely developed by these authors. The role of brain size was vastly cited [1], but detailed analysis of morphological data of the longitudinal superior fascicle, in terms of language, has not been related to speech in the context of the origin of the language, at least to our knowledge.

The longitudinal superior fascicle associates the temporal, parietal and frontal lobes in the human brain and arches around the lateral fissure [7–11]. It is the major cranial-caudal tract of the brain, with the arcuate fascicle being considered part of the longitudinal superior fascicle [8], or of the same name according to other authors [7,9–11]. Here, for simplification, the name longitudinal fascicle is used because it is a more general term.

Regardless of the given name, this structure is deeply linked to speech by associating Wernicke’s area and Broca’s area (i.e., the language area) [12,13]. The association between the longitudinal fascicle and language was, and is, defined using pathologies that compromise speech [6,13]; however, the detailed structure and function of this fascicle in humans remains controversial [10,14] and even more so in other primates [10,15].

This scenario generates uncertainty about affirmations linked to comparative analysis of the evolution of language, mainly with regard to whether the longitudinal fascicle could, hypothetically, have influenced the evolution of speech.

Interspecies study of brain structures underlying language may provide insight into the gradual evolution of the brain equipment that mediates communication [10], since data from extinct humans and non-human primates is based on hard tissues.

In this way, an investigation of the longitudinal fascicle of macaques using neuronal tracer injections showed that this structure of macaques links the posterior superior temporal gyrus (Broca’s area) with the posterior dorsolateral prefrontal cortex [16]. However, in macaques and chimpanzees the longitudinal fascicle does not reach the temporal lobe (Wernicke’s area) [10], which presents an important consequence for comparative analysis of the evolution of language.

Those studies, performed with Old World primates and humans, encompass a great phylogenetic distance for the comparative analysis of the evolution of this structure due to the absence of other kinds of primates, such as Lemuriformes, Lorisiformes, Tarsius and New World primates, with the last being a relatively poorly studied group in many areas, including phylogeny, physiology and morphology [15,17–21].

Indeed, there has been no such research on this topic involving Neotropical primates of the genus *Sapajus*. Species of this genus, interestingly and unexpectedly, have abilities for memory, cognition, social behavior and tool use that are similar to those of apes [18,22–39]. They also have large brains relative to their body mass [29,40] and high motor development [41]. Therefore, evaluating the brain morphology of this genus is an important subject for evolutionary comparative anthropology.

The behavioral similarities between *Sapajus* and apes, mainly with chimpanzees, seems to be, at least, a reasonable motivation to study the longitudinal fascicle of *Sapajus* since language is a complex behavior, and this New World primate could be expected to share identical brain structures with apes. On the other hand, anatomical studies are of great interest for generating background information to help explain the emergence of human cultural behavior, such as pollicis arrangement, bipedalism, *inter alia* [42].

For instance, *Sapajus* is capable of handling rocks to open coconuts; to use toothpicks to push food out of a pipe or to extract molasses through the orifices of a box [29,33]; to fish for termites using twigs, an activity previously only seen in chimpanzees [30]; and have been reported to display a wide array of grasping and manipulative strategies, comparable to chimpanzees and humans [29,30,33,34]. However, the main studies about capuchin language is linked to symbolism [42], putting them close to cercopithecines [43,44]. The use of the vision for communication via symbolic language is an important feature for social organization within Primates [43,44].

Therefore, the goal of the present work was to analyze, both qualitatively and quantitatively, the longitudinal fascicle of *Sapajus* sp. in comparison with that described for other primates in the literature including humans.

## Material and methods

### Subjects

A total of 24 hemispheres (12 left antimeres and 12 right antimeres) and two complete brains of *Sapajus* sp. were used in this study. The specimens, provided by the Department of Surgery, Faculty of Veterinary and Animal Science, University of São Paulo (FMVZ, USP), Brazil, derived from wild primates that experienced natural death in neighborhoods of citizens in three different states in Brazil.

Four adult males were obtained in Sete Lagoas, state of Minas Gerais, Southeast Brazil, in the 1970s. Four adult males and one adult female were obtained in Goiania, state of Goiás, Center-West Brazil (proximity of the campus of the Federal University of Goiás), 14 years ago. One adult male, one young female and one adult female were obtained in Palmas, state of Tocantins, North Brazil, seven years ago (Table 1).

**Table 1:** Specimens used this work, their location of origin, gender and estimated age.

Specimen	Local of origin	Gender	Estimated age
#1	Sete Lagoas, Minas Gerais State. Southeast Brazil.	Male	Adult from 15 to 30 years old
#2	Sete Lagoas, Minas Gerais State. Southeast Brazil.	Male	Adult from 15 to 30 years old
#3	Sete Lagoas, Minas Gerais State. Southeast Brazil.	Male	Adult from 15 to 30 years old
#4	Sete Lagoas, Minas Gerais State. Southeast of Brazil.	Male	Adult from 15 to 30 years old
#5	Goiânia, Goiás State. Center-West Brazil	Male	Adult ~25 years old
#6	Goiânia, Goiás State. Center-West Brazil	Male	Adult from 15 to 30 years old
#7	Goiânia, Goiás State. Center-West Brazil	Male	Adult from 15 to 30 years old
#8	Goiânia, Goiás State. Center-West Brazil	Male	Young ~13 years old
#9	Goiânia, Goiás State. Center-West Brazil	Female	Adult from 15 to 30 years old
#10	Palmas, Tocantins State. North Brazil	Male	Adult from 15 to 30 years old
#11	Palmas, Tocantins State. North Brazil	Female	Adult from 15 to 30 years old
#12	Palmas, Tocantins State. North Brazil	Female	Young ~9 years old

Age of such animals can be estimated by the size of the sagittal crest, size of animal or dentition, but it is very difficult to do for animals coming from nature, so only a non-precise estimation could be made.

The animals wer found by IBAMA (Brazilian Institute of the Natural Resources) and sent to the Federal University of Goiás. They had since been used in other studies and were being kept for further use in order to avoid the unnecessary sacrifice of animal lives, in compliance with international standards of bioethics and animal welfare.

The research was performed at the Federal University of Goias (UFG), Brazil (CoEP-UFG 81/2008, authorization from the IBAMA number 15275). We declare, for any purposes that it may be necessary, that the research followed the Principles for Ethical Treatment of Non-human Primates, as indicated by the guidelines of the American Society of Primatologists (ASP).

### Dissection of the intrahemispheric fiber systems of the brain of *Sapajus* sp.

The brains were stored in 10% formaldehyde solution, which was replaced after 24 hours and the brains kept for another 30 days. Klingler’s preservation method, with minor adjustments, was used to put intrahemispheric fibers on evidence for the 24 hemispheres [45,46] and two full brains, including keeping them in formaldehyde subsequent to cutting to highlight white fibers. The technique of de Castro et al. [47] was also used as a study reference.

The freezing-thawing procedure was repeated three times, which made preparation for the dissections of fiber tracts and nuclei easier by highlighting the distinction between gray and white matter. The fiber dissection technique allows three-dimensional understanding of brain anatomy, whereas Klingler’s method allows the structures that compose the internal anatomy of the fiber systems within cerebral hemispheres to be observed.

According to Klingler’s freeze-thaw method, the brains were washed for about four hours in water at room temperature. The pia mater, arachnoid, and vessels of the brains were carefully removed with small tweezers. The brains themselves were immersed in 10% formaldehyde and frozen for eight days at an average temperature of -10°C. The brains were then washed under running water for 24 hours. The freezing procedure (in 10% formaldehyde solution) was repeated three times. The brains were kept in 10% formaldehyde solution after the last freezing process.

Dissections were performed with wooden spatulas (modified from sticks and approximately 25 cm long) of different sizes and shapes appropriate to the gyri and cerebral sulci dissected. The spatulas were used for careful removal of the gray matter, after which the hemispheres were washed in running water and gently wiped and dried using paper towels. Pins or sewing needles were then used to follow the path of fibers that were coming from or going towards the prefrontal region.

The characteristics of the fiber systems in each hemisphere and in the entire brain were partially cut, analyzed and photographed, with a Canon Power Shot A520, both before and after dissection. The photos showed lateral, medial, and frontal patterns of anatomic orientation.

### Quantitative analysis

**Fig 1:**
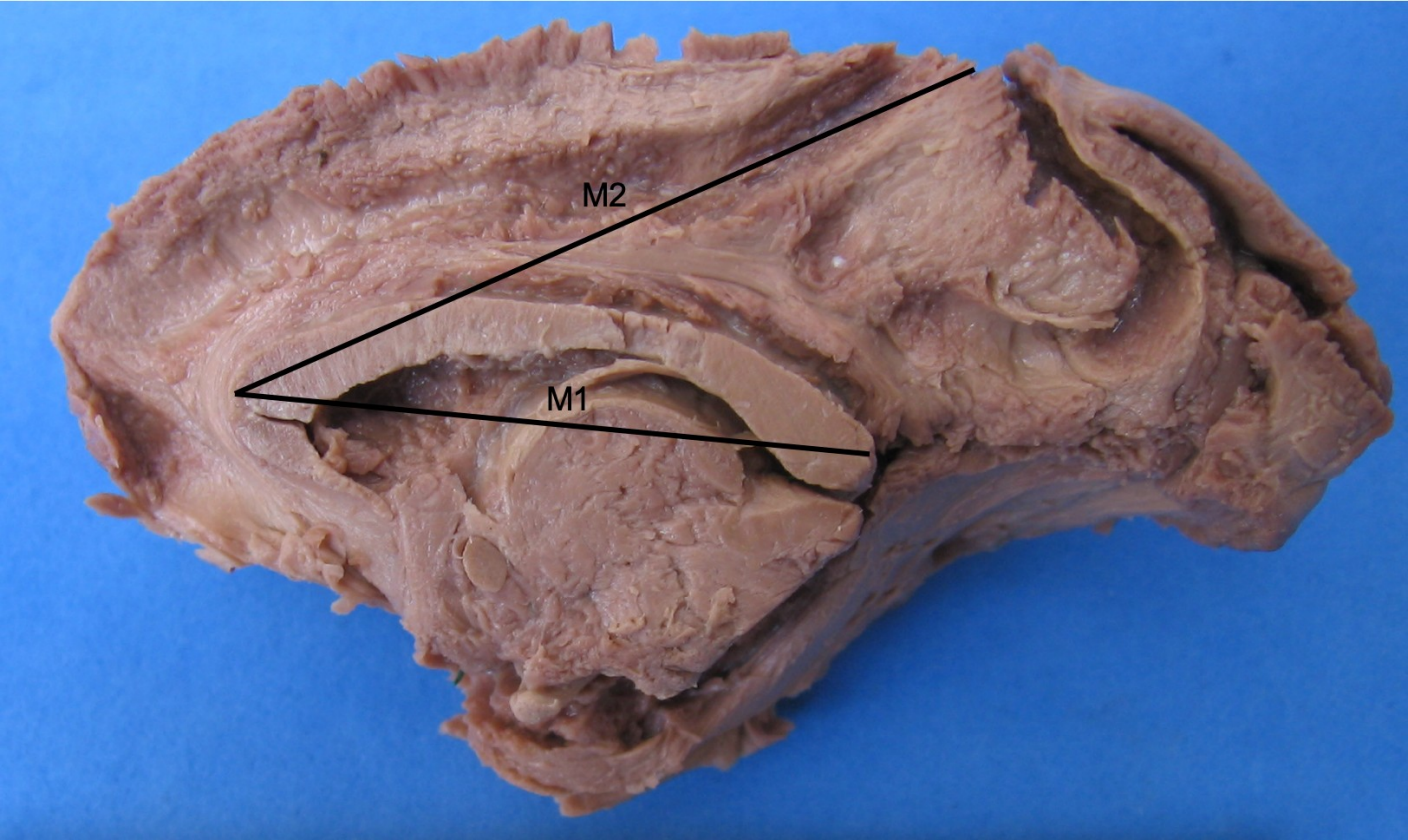
Lines indicating the obtained measurements. M1 is the measurement from the edge of the splenium to the edge of the genu of the corpus callosum. M2 is the measurement from the initial portion, coincident with the genu of the corpus callosum, to the final portion of the longitudinal fascicle [M1]. Note the deterioration of the edges of the hemisphere caused specifically by preparation for dissection of the intrahemispheric fibers. [1] Cingulate and longitudinal fascicles in the same bundle; [2] Individualized arcuate fascicle; [3] Individualized cingulate fascicle; [4] Corpus callosum; [5] Fornix; [6] Thalamus; [7] Frontal pole; [8]. Occipital pole. Specimen: Adult female. Bar = 1 cm.

Quantitative data were obtained from a straight measure of the corpus callosum, from the splenium to genu, and of the longitudinal fascicle. Due to differences in cuts of the hemispheres, which generate different positions relative to the median plane, there could be error associated with the final measurements of the caudal-cranial pole. Since the corpus callosum enters the hemisphere, these errors are diminished. A curved measurement following the structures also generates human errors. Therefore, measurements were obtained as a straight line from the edge of the splenium to the edge of the genu of the corpus callosum [M1] and a straight measure from the initial portion, coincident with of the genu of the corpus callosum, to the final portion of the longitudinal fascicle [M2], according to Fig 1. Final data were generated a the M2/M1 ratio obtained from around six photos per hemisphere to avoid variation in the protrusions of the corpus callosum and different sizes of hemispheres due to age, sex and biotype of the subjects. These methodologies do not prevent error, but diminishes it; besides, it made using scales in the photographs unnecessary, since the dimensions of the structures are directly proportional.

The M2/M1 ratio allows a verification equaling the size differences because the brain dimensions vary from young to adult and the encephalon of females is smaller than that of males of the same age. An absolute measure would generate high deviation that will not supply adequate data for comparison with other primates or for the same species.

### Statistical analysis

StatPlus:mac software (AnalystSoft Inc. v.7.1, 2020) was used for parametric analysis with average, standard deviation and T-test. Extreme values (outliers) were identified using the Q-test and *post hoc* mean comparisons were performed using the T-test.

## Results

### Quantitative analysis

**Fig 2:**
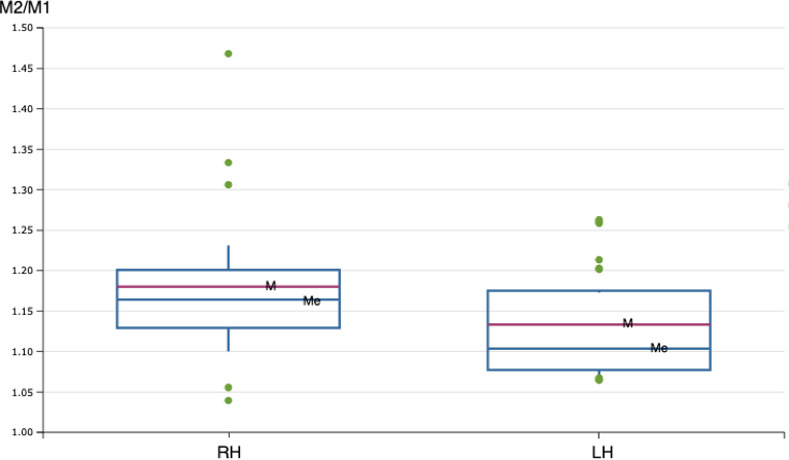
Box graphic for M2/M1 ratios plotted with mean [M], median [Me], and standard deviation [the latter delimited by upper and lower lines of the box], and outlier points shown in green color. RH is right hemisphere; LH is left hemisphere.

Means for data from the right and left hemispheres were compared by T-test to verify the accuracy of the measurements (the measurement was taken from all studied hemispheres), with the hypothesis of equality being accepted (p<0.05). The mean M2/M1 ratio was 1.1721(±0.100) for the right hemisphere and 1.131(±0.064) for the left hemisphere. This information is summarized in **Fig 2 and Table 2.**

**Table 2:** Raw data for the M2/M1 ratio of hemispheres of *Sapajus*. Absent data are due to problems with obtaining reliable data from photos.

Average M2/M1 for measures of the right hemispheres	Average M2/M1 for measures of the left hemispheres
1	1.164516129
1.055512722	1.153238547
1.039493294	1.087649402
1.221590909	1.103825137
1.163959783	1.150119142
1.468085106	1.262765957
1.333333333	1.213615023
1.306188925	1.17288444
1.142636854	1.103117506
1.18161435	1.072107765
1.175039246	1.088715953
1.146188341	1.160816327
1.1	1.260226284
1.117370892	1.258515284
1.168387609	1.201331115
1.180769231	1.202838063
1.159038014	1.151017214
1.19025522	1.130038023
1.148904006	1.10007758
1.126454616	1.077361564
1.23095429	1.064701065
1.130295763	1.064484127
**-**	1.067285383
**-**	1.070616883
**-**	1.080525883
**-**	1.07653575
**-**	1.088550984
**-**	1.094707521
**-**	1.073469388

Data were verified for normality and then the mean, median and standard deviation were calculated and shown in a box graphic (Fig 2) with all indicated (Q-test) outliers; 5/12 outlier points were verified for both hemispheres.

### Qualitative analysis

**Fig 3:**
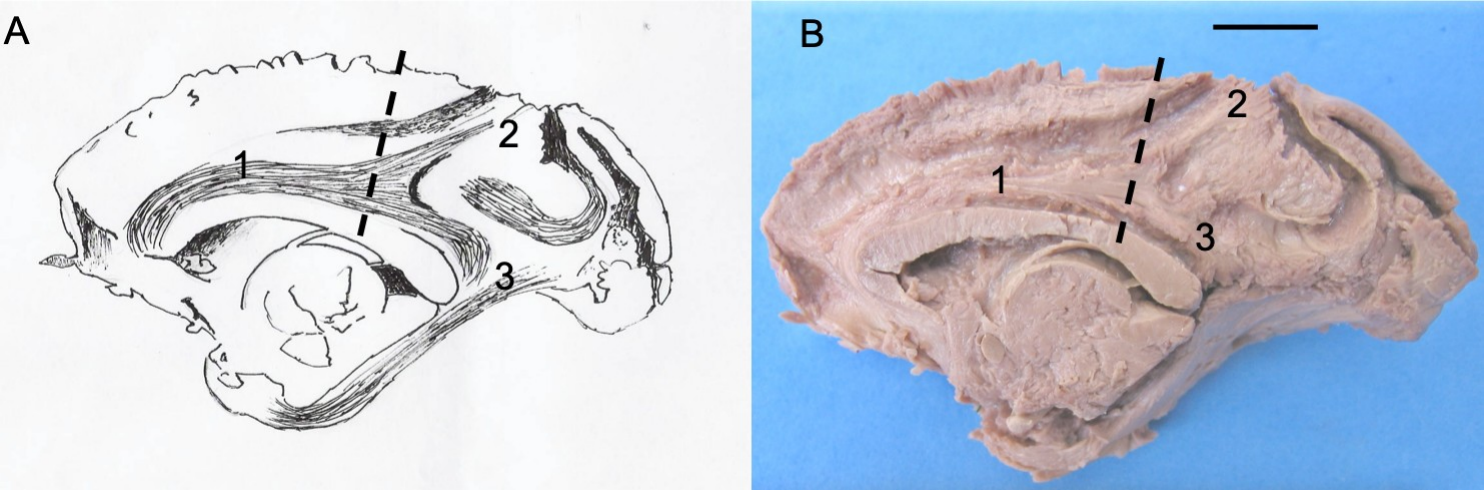
Schematic drawing (A) and photograph (B) of the right hemisphere of a specimen of *Sapajus* sp. indicating the cingulate fascicle and the longitudinal fascicle in the same bundle [1], separating approximately at level of the central sulcus [dotted line], into an individualized longitudinal fascicle [2] and an individualized cingulate fascicle [3]. [4] Corpus callosum; [5] Fornix; [6] Thalamus; [7] Frontal pole; [8] Occipital pole. Specimen: Adult male. Bar = 1 cm.

**Fig 4:**
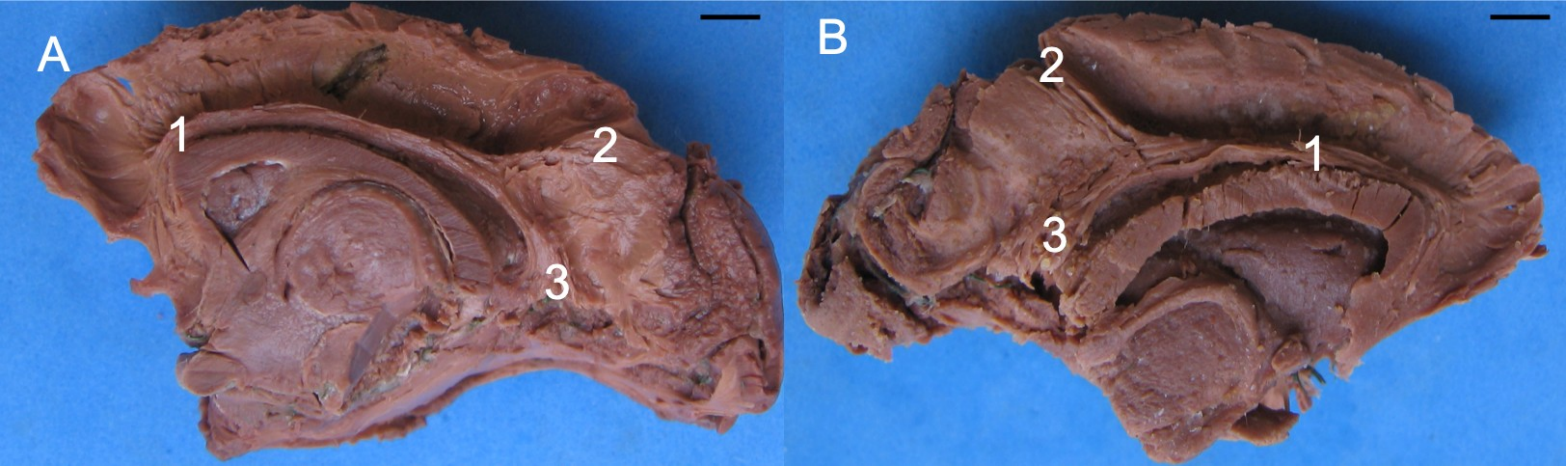
(A) Photograph of the left side of the total brain of *Sapajus* sp. illustrating the approximate locations of the cingulate-longitudinal fascicles. The longitudinal fascicle reaches the parietal lobe. Note the high lissencephaly in the frontal and occipital lobes. (B) Photograph of the medial part of a non-prepared hemisphere where the cingulate-longitudinal fascicles enter the white matter. [1] Cingulate and longitudinal fascicles in the same bundle; [2] Individualized longitudinal fascicle; [3] Individualized cingulate fascicle. [4] Corpus callosum; [5] Fornix; [6] Thalamus; [7] Frontal pole; [8] Occipital pole. Specimens: (A) adult male; (B) adult female. Bar = 1 cm.

**Fig 5:**
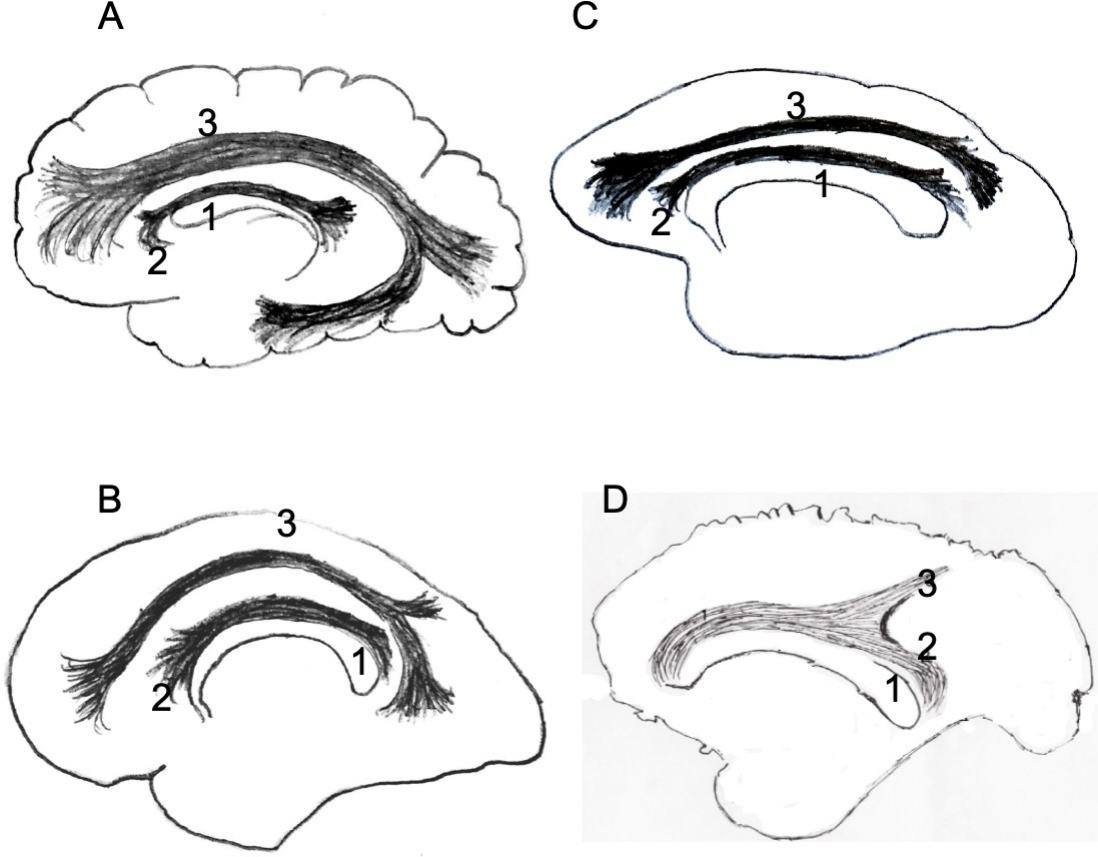
Schematic drawings of the brains of [A] human, [B] chimpanzee [representing the apes], [C] macaque and [D] *Sapajus*, indicating the [1] corpus callosum, [2] cingulate fascicle and [3] longitudinal fascicle.

An individualized longitudinal fascicle was not observed in the brain of *Sapajus* sp. (for more details see Borges et al. [15]). Indeed, the longitudinal fascicle has an origin in common with the cingulate fascicle, after which the two separate at approximately the level of the central gyrus (Figs 3A and 3B, 4A and 4B and 5).

The anatomical data do not determine whether the fibers longitudinal fascicle share the same origin or if they have different origins and use the same pathway until the separation point.

The longitudinal fascicle of *Sapajus* sp. reaches the region of the parietal lobe, as seen in medial view, but there is no evidence of fibers extending to the temporal lobe in prepared hemispheres or in cut brains (Figs 4A and 4B and 5).

**Fig 6:**
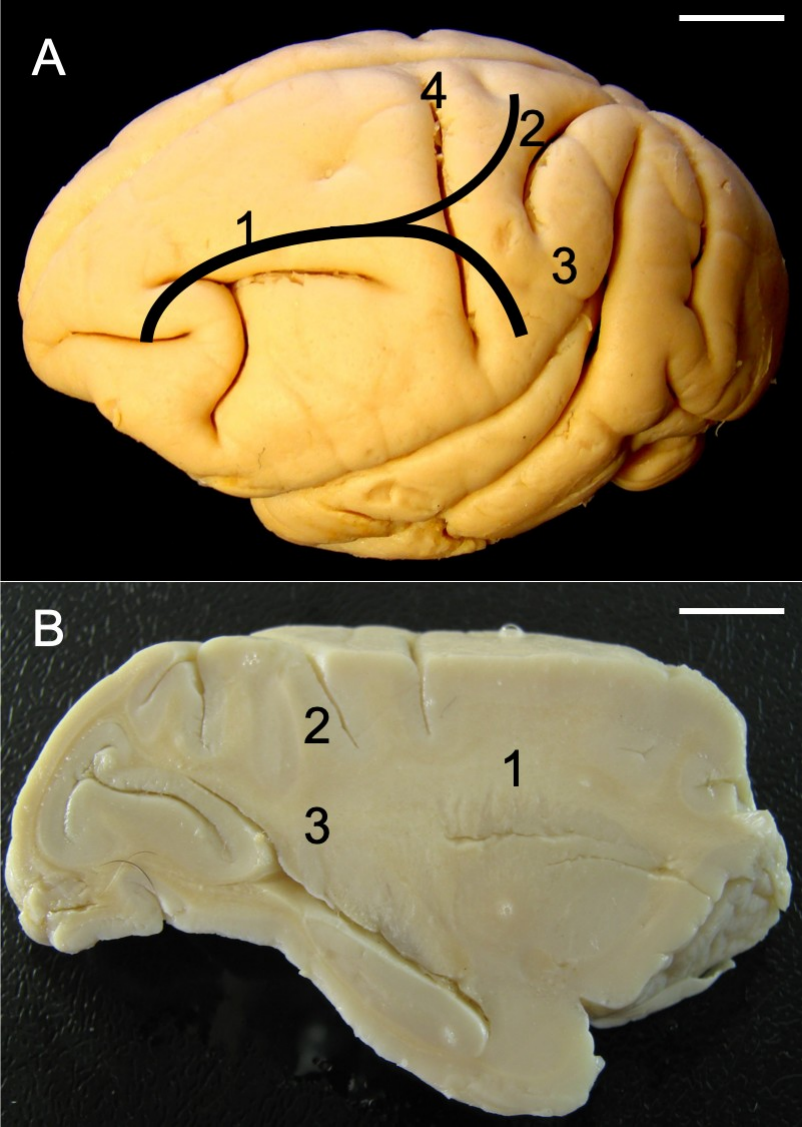
Photograph of right (lateral view) (A) and left (medial view) (B) hemispheres of *Sapajus* sp. (A) [1] cingulate and longitudinal fascicles in a same bundle; [2] individualized longitudinal fascicle; [3] individualized cingulate fascicle. Note the identical disposition, pathway and distribution of the fibers. (B) [1] Cingulate and longitudinal fascicles in a same bundle; [2] Individualized longitudinal fascicle; [3] Individualized cingulate fascicle. [4] Corpus callosum; [6] Thalamus; [7] Frontal pole; [8] Occipital pole; [9] Central sulcus; [10] Lateral sulcus; [11] Lunatus sulcus; [12] Post-central sulcus; [13] Superior temporal sulcus; [14] Vertical sulcus; [15] Occipital-temporal sulcus; [16] Calcarine sulcus. Specimens: (A) adult male; (B) adult female. Bar = 1 cm.

Mean M2/M1 ratios reveal that the longitudinal fascicle has the same comparative size in both hemispheres. Observations with the naked eye found no anatomical differences in the white fibers of the longitudinal fascicle between hemispheres (Fig 6A and 6B).

## Discussion

### Comparative anatomy of the brain and the longitudinal fascicle

Initially, it is important to consider that the Klinger’s method used in this work is a gross anatomy technique, and so human error is expected. However, some studies have indicated that this technique is the most influential method for understanding the organization of fibers linked to language, *in vivo* imaging techniques notwithstanding [48]. Other studies, however, have claimed that the limitations of this technique are mainly associated with its inability to obtain the precise volume of fibers and their penetration into gray matter, which can be provided by a 3D technique [49].

On the other hand, it has been proposed that both techniques be used to obtain better 3D images [50], while a recent study using the Klinger’s method showed the same disposition of the longitudinal fascicle as obtained by imaging methods [51].

Regardless of the method, general studies of the brain of *Sapajus* [40] have indicated interesting and unexpected evidence of how it sustains complex behavior on par with chimpanzees [17,18,20,22,27,30,52–57].

A detailed study of gyrencephaly and curvature/sinuosity of the main gyri and sulci demonstrated that the brain of *Sapajus* is lissencephalic in the convex part of the frontal and occipital lobes, while parietal and temporal lobes are more gyrencephalic [40] (Fig 4A).

In general, the gyri of the brain of *Sapajus* are less sinuous than those of baboons, chimpanzees and humans, which is consistent with data that show increased complexity of sulci and gyri from prosimians to humans [58]. However, lateralization, which is a characteristic of derivative brains, at least in Sylvian’s fissure, was observed in these capuchins [59].

The encephalization index for *Sapajus*, which is a measure of relative encephalon size and is an indication of cognition, has been reported as 2.21 (±0.31) [40] and 4.8 [60,61], the latter of which is greater than the 2.2–2.4 for chimpanzees [60].

In general, both neural [15,40] and muscular [17,18,20] comparative studies of *Sapajus*, baboons, chimpanzees and humans place *Sapajus* closer to baboons.

Another similarity is that Cercopithecines (*Macaca*) and *Sapajus* have identical symbolic language, using threat faces and fear grimaces, putatively because a convergence from a common ancestral or because a functional need in social behavior [43,44].

These seems contradictory with regard to the complex behavior presented by *Sapajus*. A plausible explanation, in opposition to anatomical data, could be found in the histology of cortical structure. This includes the disposition, number and density of cells, at least [15,40]; and the volume of white matter, which increases neural connectivity, and is relatively larger in humans than in other primates [62], followed by gorillas and *Sapajus* [63], rather than chimpanzees, for instance.

This information could, at least partially, explain the intelligence of *Sapajus*. However, such data are not, or are scarcely, available, not only for *Sapajus* but for other primates as well, perhaps with the exception of chimpanzees. Plus, the study of white matter, in the present and other works, has not produced significant data to justify the cognition of *Sapajus* [15].

Following discoveries regarding the comparative anatomy of the neural system of *Sapajus*, the results of the present work reveal a greater degree of similarity with macaques [10,15,40] than with chimpanzees and humans [17,22]. Studies comparing the gross anatomy of brain structures have indicated more similarities between *Papio* (baboons) and *Sapajus* in relation to the main sulci and gyri, considering only *Sapajus*, Old World primates and apes (for more details see Pereira-de-Paula [40]), whereas no comparisons with other New World primates have been presented. According, the degree of gyrencephally is greater in *Papio* than *Sapajus* and greater than both in apes. However, the convex aspect of the brain and the lesser complexity of general brain morphology in *Sapajus* and *Papio* permit the conclusion of high similarity between these primates.

In quantitative terms, the relative dimensions of the longitudinal fascicle of *Sapajus* (i.e., M2/M1) demonstrates similarity between both sides (p<0.05, T-test). The M2/M1 ratio (see Material and Methods section) diminishes human error when only considering raw values, because the errors are divided. In other words, the expected greater human error disappears as a in function of the ratio. Plus, M2/M1 shows, in the same proportion, the size and differences among brains (for more details see [18–20,40]).

To our knowledge, this kind of measurement has not been verified for other primates, however, anatomical data for Old World primates and apes indicate bilateral symmetry for the longitudinal fascicle in humans, chimpanzees and macaques, at least before it to divides to the temporal and parietal lobes in humans and just to the parietal lobe for the other cited primates [10].

However, and it must be emphasized, the union of cingulate and longitudinal fascicles [15] is evident in *Sapajus* and has not been reported or shown for any other primates. As this study of the longitudinal fascicle is, to our knowledge, unique for New World primates, *Sapajus* could be considered as a link between primitive primates, New World primates and Old World primates, considering general brain evolution of the longitudinal fascicle. However, more studies must be performed on primitive primates, because New World primates could provide better morphological comparisons for further knowledge about the evolution of language.

The data obtained here for the longitudinal fascicle of *Sapajus* seems interesting, at least because the unexpected surprisingly high cognition, tool use and manual skills presented by *Sapajus* would imply a similar longitudinal fascicle for chimpanzees, but this was not observed in the present work.

This feature of the longitudinal fascicle in *Sapajus* seems to be a primitive feature, because the observed tendency is that intrahemispheric fascicles evolved for individualization in the direction of humans with greater prefrontal white matter than other primates [64,65].

Gross anatomy of the longitudinal fascicle, along with other neural studies of *Sapajus*, are not sufficient to explain its high cognition. Nor are there plausible phylogenetic clues due to the general lack of intrahemispheric morphological data for Primates.

Indeed, there is no information about intrahemispheric fascicles for other New World primates, and only limited data about Old World primates, with emphasis on macaques and, among apes, chimpanzees. This is not sufficient to construct a valid and robust explanation of the evolution of the longitudinal fascicle and, therefore, for the functions linked to this structure, such as language.

Recent studies about intrahemispheric fascicles have been performed mainly with imaging technologies, which was not the case for *Sapajus*. However, the present study revealed that the structure of the core of the fascicles of *Sapajus* are evident and positive using Klingler’s method [15] and could serve as a useful basis for future 3D imaging studies, since some studies have used both anatomical and *in vivo* techniques to obtain better image data [49–51].

Detailed observations of structures of the brain of *Sapajus* will require imaging techniques, since such techniques can help to determine if parts of the longitudinal fascicle that have been demonstrated for other studies, including humans, exist in *Sapajus* [66].

The longitudinal fascicle of humans follows a regular pathway, but variation in its final target—temporal and parietal lobes—can occur in the same or different individuals [66], as shown by imaging and anatomical methods [49–51].

Evidently, in evolutionary terms, humans have a greater area of connections associated with speech, including temporal and parietal lobes. This is not, however, the case for macaques and chimpanzees [10], nor for *Sapajus*, in the present work, which possesses a more symbolic communication in social behavior [43,44].

Although longitudinal and cingulate fascicles were individualized in humans using imaging techniques, which also generated two different names for the longitudinal fascicle—I and II superior longitudinal fascicle [10]—this difference was also observed in gross dissection [51].

This does not mean that the temporal lobe does not receive fibers linking it and the frontal lobe, and it is plausible to consider that white matter fibers link these regions without generating an evident fascicle, at least for *Sapajus*, considering the Klingler’s method.

An important issue is verifying the united cingulate and longitudinal fascicles, and determining whether the fibers arise from the same or different origins in the frontal lobe of *Sapajus*, because the frontal origin of the longitudinal fascicle seems to be regular for macaques and chimpanzees [10].

For human studies, it has been historically [6,67–72] and recently [62,63,73] accepted that language involves a vast cerebral area and brain connections associating all lobes and different areas of these lobes.

The fact that language embraces many cerebral areas does not indicate that specific anatomical regions do not take part in language. Instead, they are important as intermediaries for the final activity, which is the emission of coordinated sound for speech or signal for language.

As *Sapajus* and macaques share an identical symbolic language [43,44], anatomical data about the lack of a longitudinal fascicle association with the parietal lobes seems not to interfere with the visual aspects of symbolic language linked to visual perceptions.

Accordingly, anatomical studies of the longitudinal fascicle in Primates could offer insights into the evolution of language in relation to areas connected to this complex functional system [45], such as showing new areas that increased in Primates to become areas that allow effective language in humans.

This kind of studies could indicate a more logical way to understand the evolution of cognition based on positive data from anatomical, imaging or dissection methods.

## Conclusions

Anatomical data from the present study reveals a longitudinal fascicle in the brain of *Sapajus* that is primitive in relation to other studied primates, such as macaques and chimpanzees. However, *Sapajus*, macaques and chimpanzees share a longitudinal fascicle that does not reach the temporal lobe in the way that it does in humans, putatively because symbolic language is more usual for these species.

Anatomy of the longitudinal fascicle, and other morphological data obtained from *Sapajus* in other works, are in contradiction with the cognition observed in these primates.

More primitive than that of macaques and chimpanzees, the longitudinal fascicle of *Sapajus* presents a different bundle organization with the union of the cingulate gyrus and the longitudinal fascicle until approximately the level of the central sulcus. However, studies using imaging techniques are necessary to validate this finding for *Sapajus*, as are additional studies about the longitudinal fascicle in New World primates using cytoarchitecture analysis.

## Supporting information

S1 Fig (JPG)

S2 Fig (JPG)

S3 Fig (JPG)

S4 Fig (JPG)

S5 Fig (JPG)

S6 Fig (JPG)

S7 Fig (JPG)

S8 Fig (JPG)

S9 Fig (JPG)

S10 Fig (JPG)
