# Supplementary figures and images for: Gross anatomy of the longitudinal fascicle of Sapajus sp
Source: PLoS One. 2021 Jun 24;16(6):e0252178. doi: 10.1371/journal.pone.0252178 (PMC8224874; doi:10.1371/journal.pone.0252178)

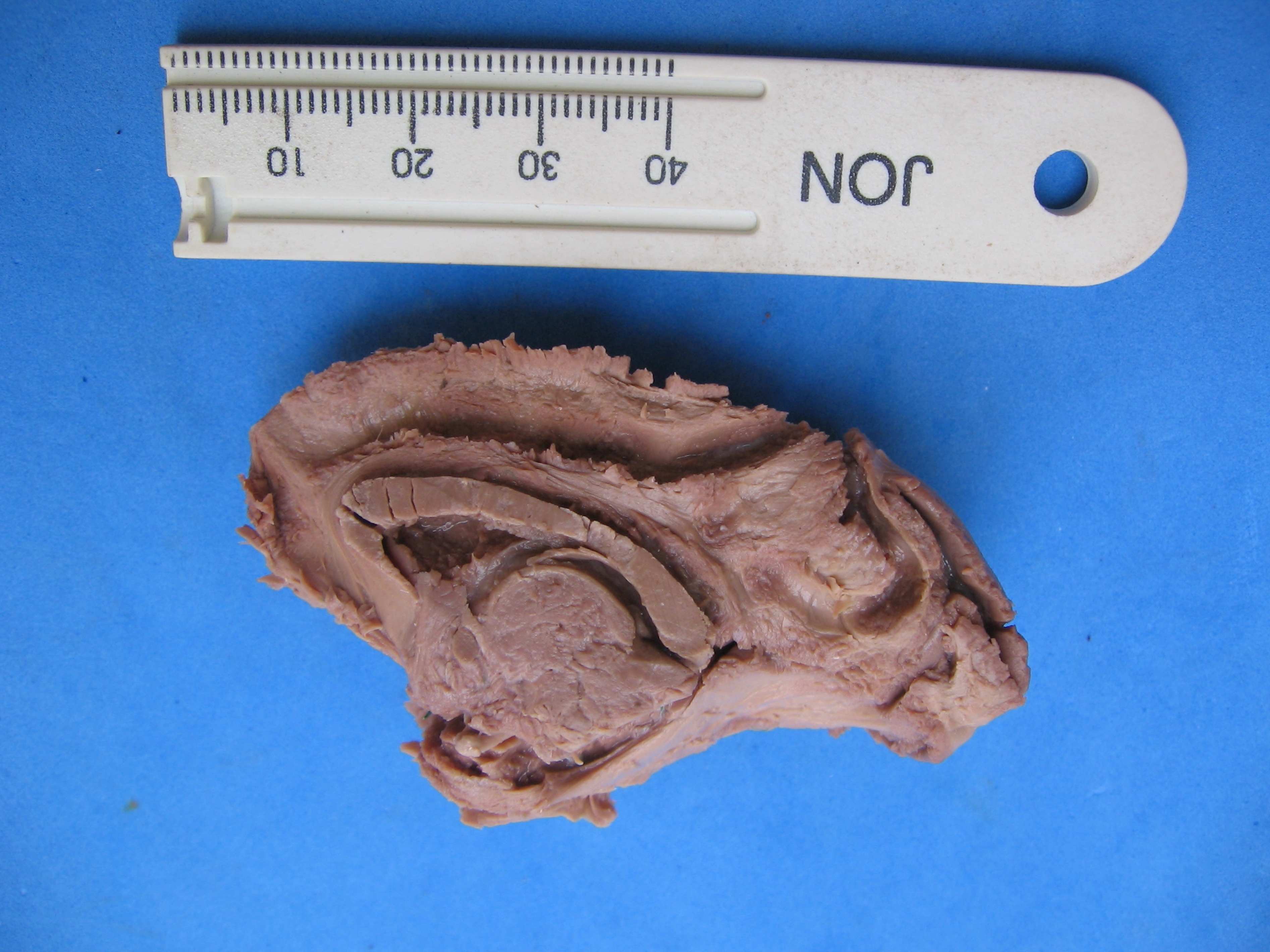

Supplement: S3 Fig — (JPG) [file pone.0252178.s003.jpg]

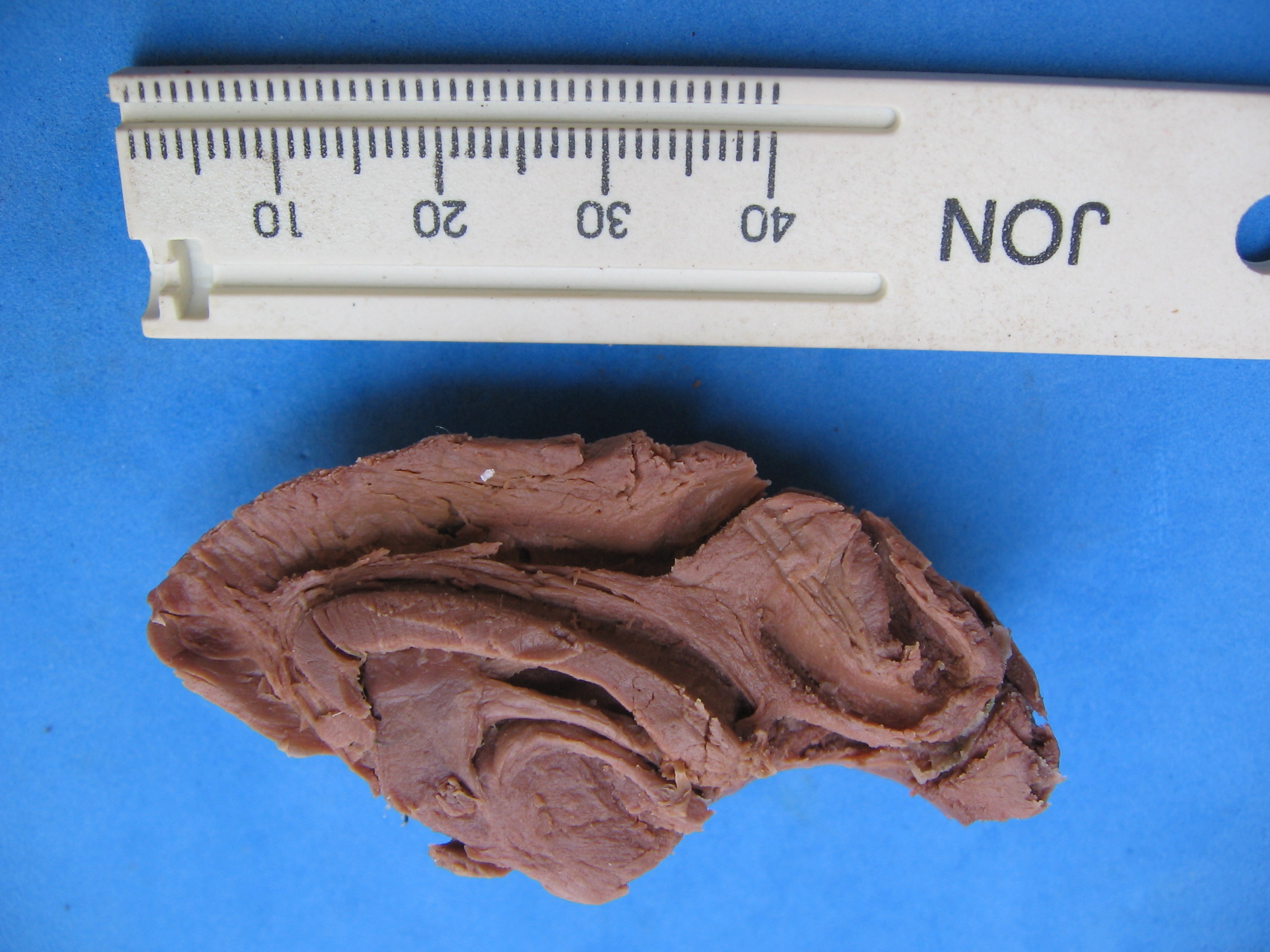

Supplement: S4 Fig — (JPG) [file pone.0252178.s004.jpg]

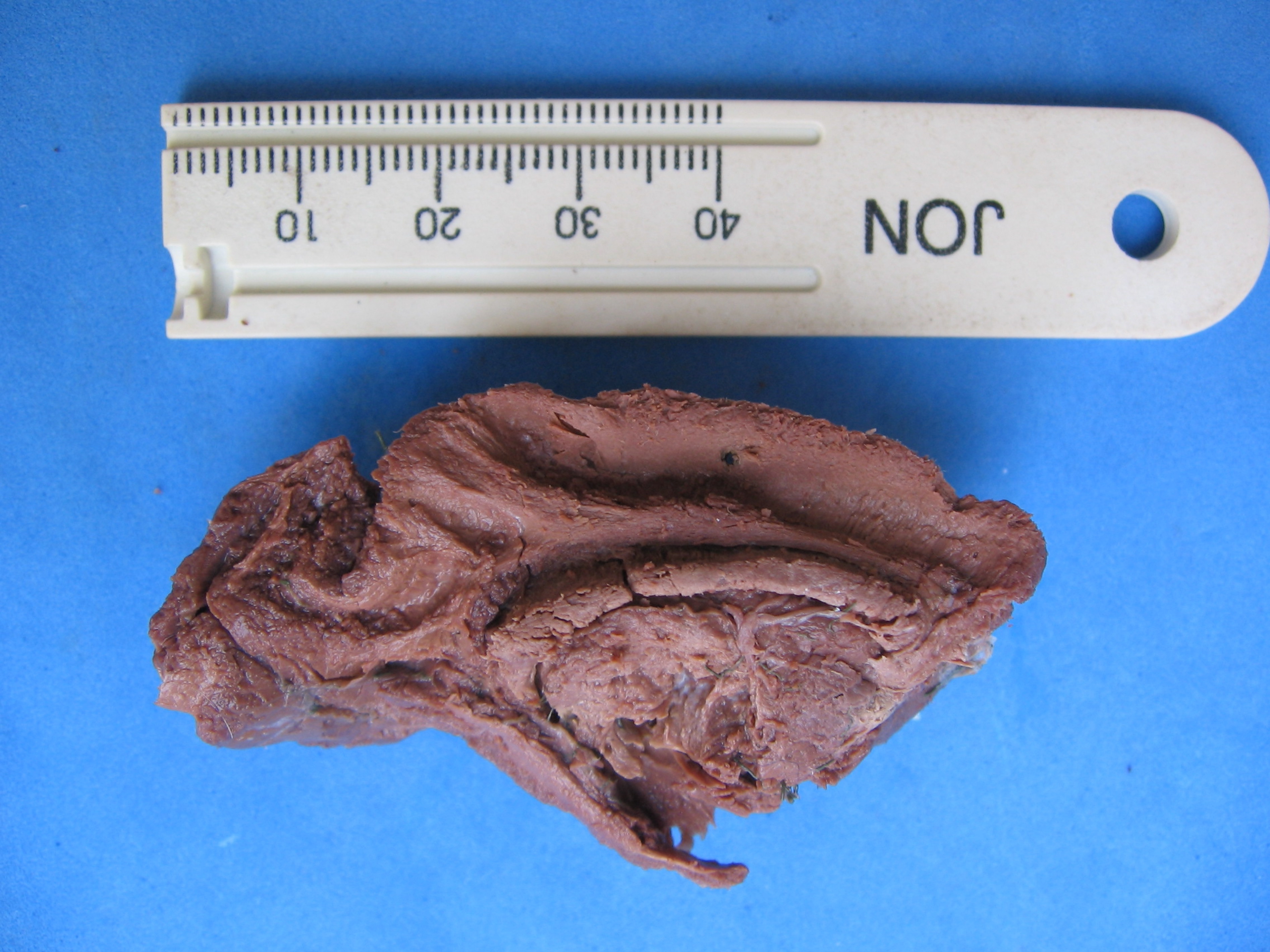

Supplement: S5 Fig — (JPG) [file pone.0252178.s005.jpg]

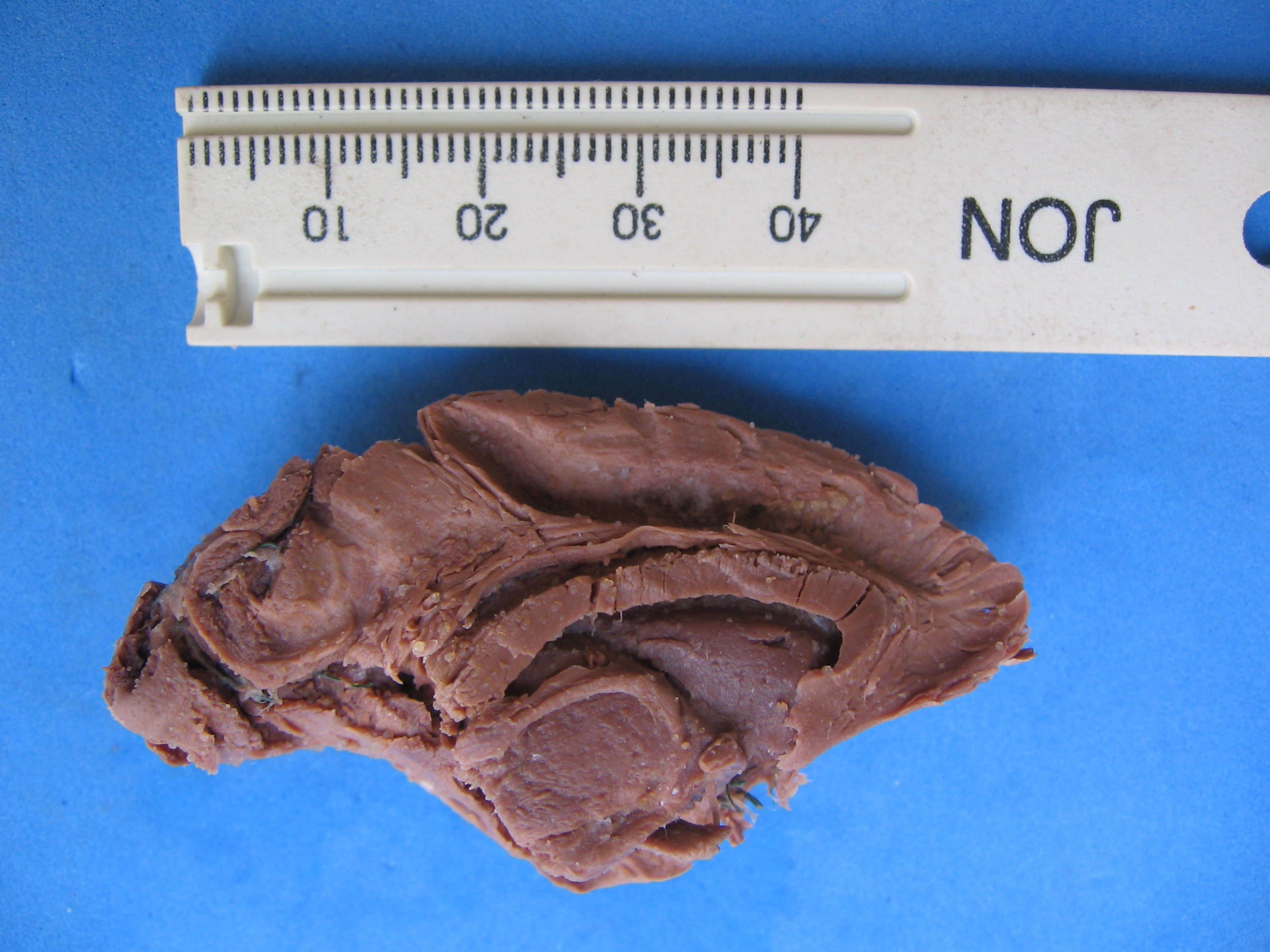

Supplement: S6 Fig — (JPG) [file pone.0252178.s006.jpg]

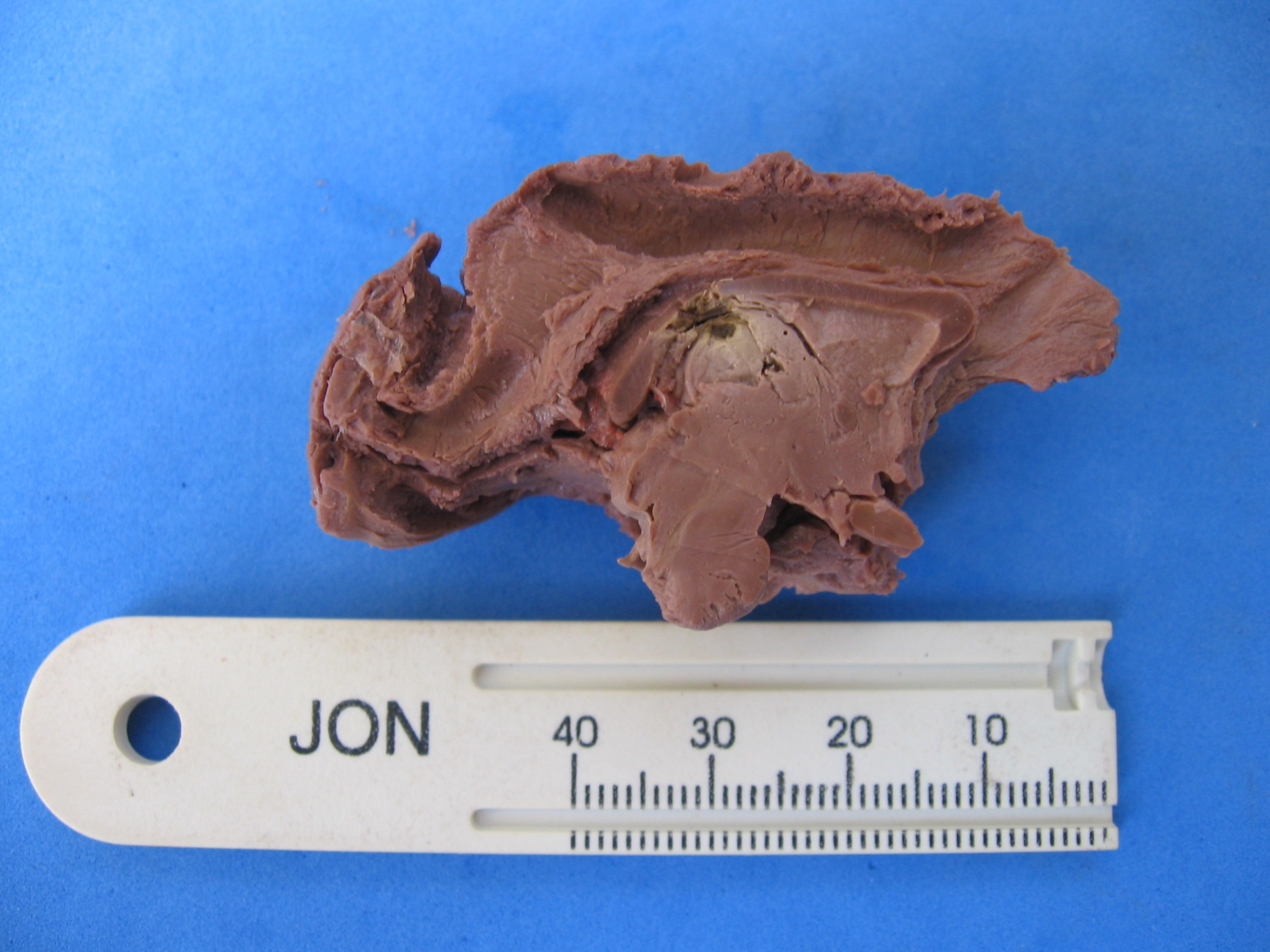

Supplement: S7 Fig — (JPG) [file pone.0252178.s007.jpg]

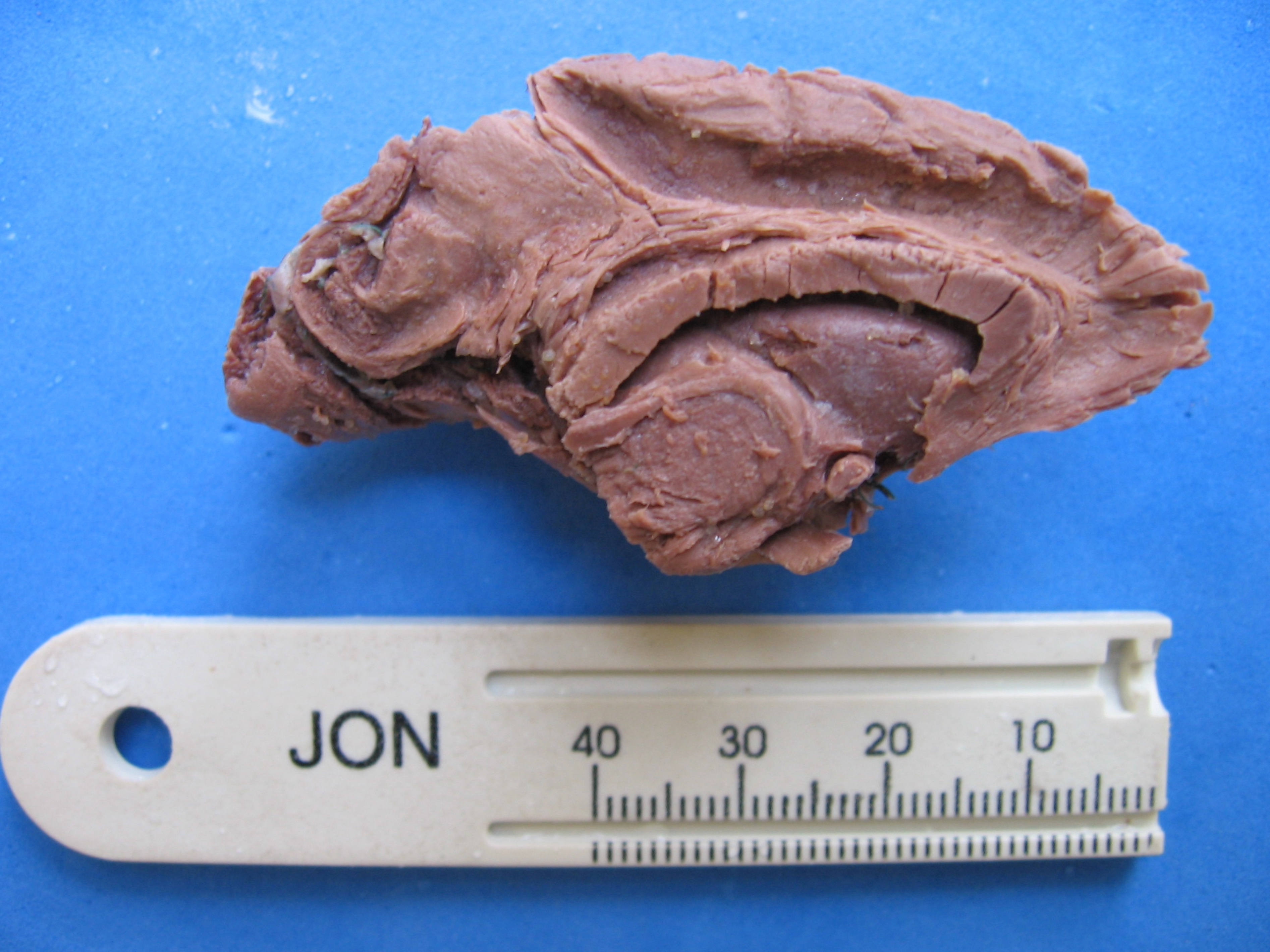

Supplement: S8 Fig — (JPG) [file pone.0252178.s008.jpg]

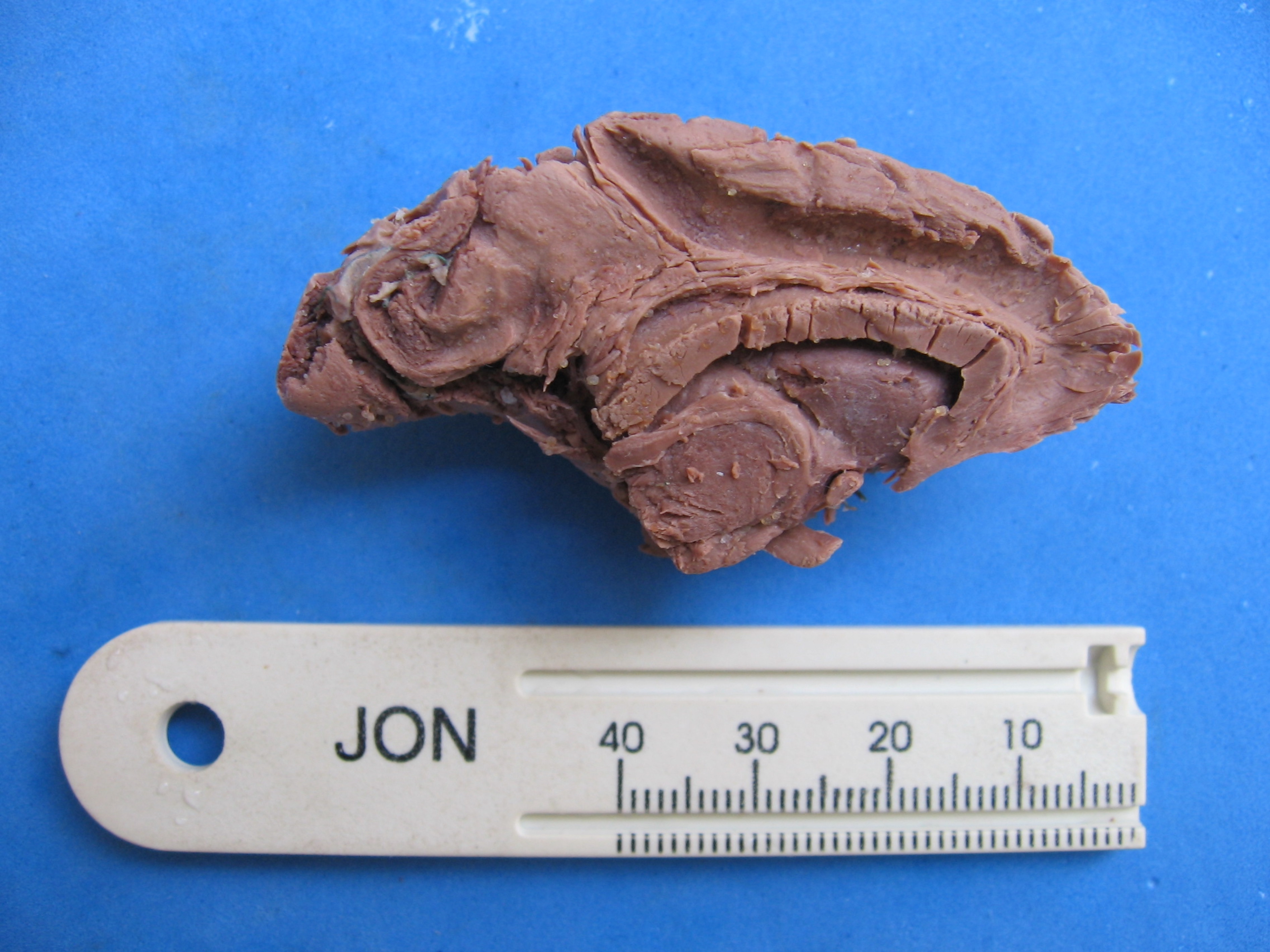

Supplement: S9 Fig — (JPG) [file pone.0252178.s009.jpg]

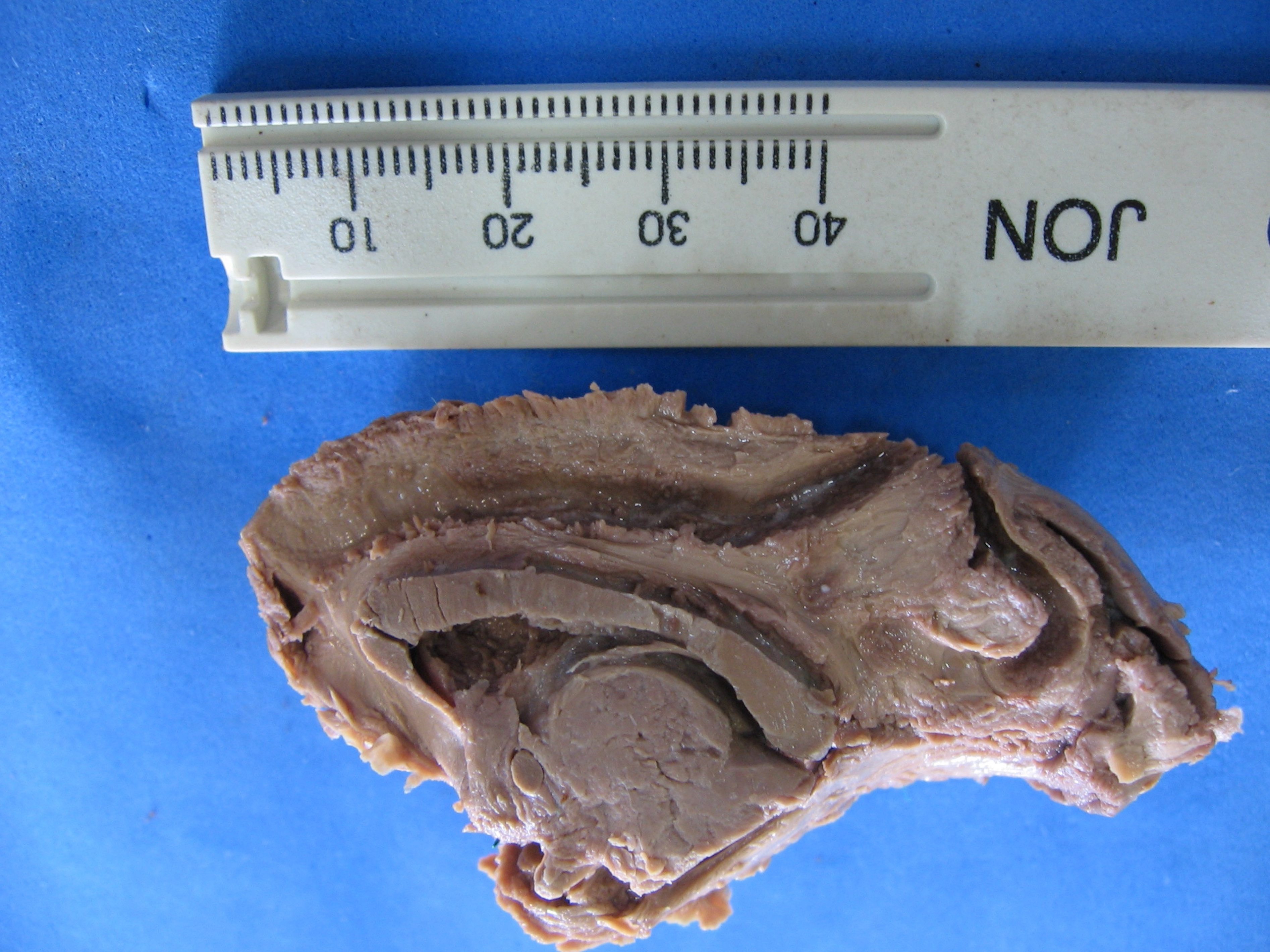

Supplement: S10 Fig — (JPG) [file pone.0252178.s010.JPG]
